# A Text-Book of Bacteriology

**Published:** 1896-10

**Authors:** 


A Text-Book of Bacteriology. By George M. Sternberg, M. D., LL. D., Sugeon-General U. S. Army. Illustrated by heliotype and chromo-lithograpic plates and 200 engravings. New York: William Wood & Company. 1896.

The manual of bacteriology, by the same author, published in 1893, had so large a sale and was so well received that it seemed advisable to issue a new work embodying all of the practical matter contained in the manual, together with such advances as have been made since the former date, and omitting all those portions of the older work which were in any respect theoretical, or purely scientific without practical application; in other words, to make a book especially fitted for the use of the practitioner and student of medicine. This the present volume accomplishes, and, with added illustrations, it is without exception the finest text-book of bacteriology published in any language.

The work is divided into four parts, Part I. dealing with the classification of morphology and general bacteriological technology; Part II. with general biological characters; Part III. with pathogenic bacteria, and Part IV. with the saphrophytes. It will be thus seen that the author has excluded from this text-book the detailed description of the nonpathogenic bacteria and the extensive bibliography found in the manual which was reviewed in the November, 1893, number of the Journal. The book is handsomely printed, the plates beautiful and the illustrations exceedingly well executed. W. C. K.

